# Treating Pediatric Asthma According Guidelines

**DOI:** 10.3389/fped.2018.00234

**Published:** 2018-08-23

**Authors:** Riccardina Tesse, Giorgia Borrelli, Giuseppina Mongelli, Violetta Mastrorilli, Fabio Cardinale

**Affiliations:** Allergy, Immunology and Pediatric Pulmonology Unit, Ospedale Pediatrico Papa Giovanni XXIII, Bari, Italy

**Keywords:** asthma, pharmacology, guidelines, asthma management, children

## Abstract

Asthma is a common chronic inflammatory disorder of the lower respiratory airways in childhood. The management of asthma exacerbations and the disease control are major concerns for clinical practice. The Global Strategy for Asthma Management and Prevention, published by GINA, updated in 2017, the British Thoracic Society/Scottish Intercollegiate Guideline Network, revised in 2016, the National Institute for Health and Care Excellence asthma guideline consultation, available in 2017, are widely accepted documents, frequently implemented, with conflicting advices, and different conclusion on asthma definition and treatment. An International Consensus on Pediatric Asthma was carried out in 2012 by a Committee with expertise in the field, to critically review differences on current guidelines. In addition, the specific issue of treating severe and difficult asthma has been recently highlighted throughout the International European Respiratory Society/American Thoracic Society guidelines on severe asthma. The aim of this paper is to describe conventional treatments and some new therapeutic approaches to pediatric asthma according to guidelines, highlighting key aspects, and differences on proposed clinical recommendations for asthma management. Age specific therapy are proposed in steps, according to clinical severity and the level of disease control. If control is not achieved within 3 months, stepping-up should be considered; otherwise, if control is achieved after 3 months, stepping down may be considered. The most used drug classes of asthma medications are beta-2 adrenergic agonists, corticosteroids, and leukotriene modifiers. Intramuscolar triamcinolone has been used for severe asthma treatment. Chromones and xanthines have been extensively used in the past, but they have shown limits related to their efficacy and safety profile. Omalizumab, a monoclonal antibody against IgE, is an immunomodulatory biological agent, used as new drug in patients with confirmed IgE-mediated allergic asthma, only for patient's specific range of total IgE level. There are low evidences in the efficacy of metotrexate, as well as macrolide antibiotics in children with asthma. Antifungal agents are also not recommended in asthmatic patients. Non-pharmacological measures that may improve patient's quality of life should also be attempted. We conclude that treatment decisions on childhood asthma management should be critically made, pondering the differences suggested by agreed international consensus documents.

## Introduction

Asthma is a heterogeneous chronic airway disease very common in childhood, usually characterized by respiratory symptoms including wheeze, breathlessness, chest tightness and cough, together with variable expiratory airflow obstruction (1, 2). There is widespread concern about symptom control in asthmatic patients, as well as risk of adverse outcomes into clinical practice. It is therefore not surprising that several guidelines are available to support health care professionals on asthma management of children and adults.

The Global Strategy for Asthma Management and Prevention (GINA) and the British Thoracic Society (BTS)/Scottish Intercollegiate Guideline Network (SIGN) are widely accepted documents, released in the USA and in Europe, respectively. Since 2002 the GINA Science Committee was established to review published research on asthma. Expert leaders in adult and pediatric asthma research, regularly meet with the American Thoracic Society (ATS) and European Respiratory Society (ERS) international conferences, to critically review asthma-related scientific literature. The major revision of GINA has been updated in 2017, suggesting recent conclusions on asthma definition and treatment (3).

The first BTS guideline dated back to 1990 and the SIGN guidelines to 1996, but in 1999 the two Society jointly produced new asthma guideline, updated annually, until 2016, and made available on websites (4).

Both guidelines are frequently implemented, providing recent updates recommendations for asthma management and prevention, with conflicting advices and different conclusion on asthma definition and treatment (5, 6). In 2015 the United Kingdom (UK) National Institute for Health and Care Excellence (NICE) also released a consultation document for diagnosis and monitoring of asthma, and later, on December 2016, for the management of chronic asthma, including cost benefit analysis that were not considered in the BTS/SIGN guidance (7).

An International Consensus on Pediatric Asthma was carried out in 2012 by a working Committee with expertise in the field (8), to critically review differences on the current abovementioned International guidelines, but also including other reports like the Australian Asthma Management document and the Japanese Guideline for Childhood Asthma (9, 10).

Non pharmacological measures that may improve quality of life and reduce symptoms in asthmatic people should be attempted, particularly in children. They include avoidance of exposure to environmental tobacco smoke or cessation of smoking among adolescents, avoidance of food or drug triggers, in people sensitive to them, avoidance of indoor and/or outdoor pollution and irritants (11, 12). Weight loss in overweight patients should be advised; breathing exercise programmes, following physiotherapist methods should also be encouraged.

However, pharmacotherapy represents the fundament of asthma treatment in adults and children. The international reports define the principles of pharmacological asthma management and indicate age specific treatments in steps, according to clinical severity and the level of disease control, which is determined by the interaction between the patient's genetic background, the ongoing treatment, environment, and psychosocial factors. Asthma severity is assessed retrospectively from the level of treatment required to control symptoms and exacerbations, that may change over months.

Asthma phenotypes have been defined based on specific pathologic features, demographic and clinical patterns or therapy responses and personalized phenotype-guided asthma treatments are under investigation, with the acknowledgment of the patient's adherence to therapy issues (13).

This review gives a practical perspective on the most used drugs and the basic steps of asthma management in children and adolescents, based on current international guidelines recommendations, highlighting the key aspects and differences of these documents.

## Categories of asthma medications and step-wise strategies of treatment

The pharmacological options for treatment of asthma include, according to their use, reliever medications, which are drugs that allow relief of symptoms within few minutes, during worsening asthma or exacerbations, also used for prevention of exercise-induced bronchoconstriction; controller medications, that are used for maintenance treatment: they control symptoms and reduce airway inflammation and future risks of exacerbations; add-on therapies, proposed for patients with severe persistent asthma symptoms and exacerbations, despite treatment with high dose controller medications.

The most used drug classes of asthma medications are beta-2 adrenergic agonists, corticosteroids, and leukotriene modifiers, usually montelukast.

A stepwise approach for pharmacotherapy management in asthmatic patients has been proposed (Table 1). Treatment starts at the step most appropriate to the initial severity of asthma. If control is not achieved within 3 months, stepping-up should be tried, after reconsidering adherence to therapy, environment factors, and associated co-morbidities; otherwise, treatment step down may be attempted once good asthma control and the patient's lowest effective level of treatment has been found and maintained for about 3 months. Each recommendation has been assessed for adults adolescents (over 12 years) and children (5–12 years, and under 5 years) in all guidelines (3, 4).

**Table 1:** Stepwise pharmacotherapy management in asthmatic children.

	**STEP 1**	**STEP 2**	**STEP 3**	**STEP 4**	**STEP 5**
**Reliever therapy**	As-needed SABA		As-needed SABA or low dose ICS/ LABA		
**Controller therapy**		Low dose ICS	Low /medium ICS/LABA	Medium/high ICS/LABA	Add-on treatment (omalizumab)
**Other common controller options**	Low dose ICS	LTRA	Medium/high dose ICS Low ICS + LTRA	High dose ICS + LTRA	Low dose OCS

SABA, short-acting beta_2_-agonist; ICS, inhaled corticosteroids; LABA, long-acting beta_2_-agonist;

*LTRA, leukotriene receptor antagonists; OCS, oral corticosteroids*.

### Medications used for rapid relief of symptoms

At present, **Step 1** treatment is with as-needed inhaled short-acting beta2-agonists (SABAs) alone, commonly salbutamol. SABAs are used for acute relief of asthma symptoms, mainly in patients with occasional daytime symptoms and with normal lung function. Inhaled anticholinergic agents, usually ipratropium, are second-line relievers (14); they are less effective than SABA, but may have synergistic effects when added to SABA during severe exacerbations in reducing patients hospitalization (3, 4).

In adults, oral SABA or short-acting theophylline are potential alternatives to SABA, however, they have a slower onset of action than inhaled SABA and a higher risk of side-effects, and they are not recommended in children (15).

### Medications used for long-term asthma symptoms control

#### Inhaled corticosteroids (ICS)

More frequent symptoms or the presence of any exacerbation risk factors indicate that regular controller treatment is needed. For long term asthma control in children, a maintenance treatment with therapeutic doses of ICS in addition to as-needed SABA, should be considered. Regular low dose ICS improve asthma symptoms and lung function, decrease need for additional medication and hospital admission. In the international reports this combination of drugs is the first option of treatment included into **Step 2** (3, 4). ICS differ in potency and bioavailability; beclomethasone dipropionate (BDP) and budesonide have approximately equivalent effects in clinical practice, although there may be some variations using different delivery devices. Fluticasone propionate and mometasone appear to provide the same clinical activity, compared to BDP and budesonide, at half the dosage. The initial dose of ICS should be appropriate to the severity of disease (Table 2). In children and adolescent, the starting ICS dose will usually be less or equal 200 micrograms BDP or equivalent per day, given initially twice daily (except ciclesonide proposed once daily). More than 200–400 μg BDP or equivalent would be considered a pediatric moderate dose, and more than 400 μg a pediatric high dose. The dose of ICS should then be titrate the to the lowest effective dose at which control of asthma is maintained. There is an increasing evidence showing that, at recommended doses, ICS are also safe and effective in young children with asthma (16).

**Table 2:** Low, medium, and high inhaled corticosteroids doses in asthmatic children and adolescents.

**Inhaled corticosteroid**	**Total daily dose (mcg)**		
	**Low**	**Medium**	**High**
**SCHOOL AGED CHILDREN**			
Beclometasone dipropionate	100–200	200–400	>400
Budesonide	100–200	200–400	>400
Ciclesonide	80	80–160	>160
Fluticasone propionate	100–200	200–400	>400
Mometasone furoate	110	220–440	>440
Triamcinolone acetonide	400–800	800–1,200	>1,200
**ADOLESCENTS**			
Beclometasone dipropionate	200–500	500–1,000	>1,000
Budesonide	400–200	400–800	>800
Ciclesonide	80–160	160–320	>320
Fluticasone propionate	100–250	250–500	>500
Mometasone furoate	110–220	220–440	>440
Triamcinolone acetonide	400–1,000	1,000–2,000	>2,000

However, long term follow-up studies have demonstrated some effect of the chronic ICS use at intermediate-high doses with growth retardation in pre-pubertal children in the first years of treatment, and the reduction in adult final height (17–19). Poorly-controlled asthma itself may affect patient's growth (20). Therefore, after symptoms control has been achieved, ICS therapy should be gradually tapered to the lowest effective dose.

Clinical adrenal suppression has also been described in a small number of children who had been treated with ICS (21). The duration of ICS treatment that may expose a child at risk of clinical adrenal insufficiency is unknown but is likely to occur at high ICS dose per day (at ≥800 μg BDP per day or equivalent). Tests of adrenal function such as the low-dose adrenocorticotrophic hormone (ACTH) test, may be useful in predicting clinically relevant adrenal insufficiency in a child using high-dose ICS, but it is unknown how frequently would need to be repeated in children.

Moreover, the use of extra-fine (diameter <2 μm) vs. fine particle BDP administered as mono-therapy or in combination with LABA, have shown reduction in exacerbation rates in severe asthmatic patients, in a meta-analysis of observational studies (22). Corticosteroid insensitivity is also another specific issue under investigation: molecular mechanisms including glucocorticoid receptor alteration, have been proposed (23).

For patients with persistent symptoms or recurrent exacerbations despite the use of low dose ICS, may be considered a step up, after checking for medication adherence, inhaler technique, continuous allergen exposure and comorbidities such as rhinosinusitis, gastroesophageal reflux, obesity, and obstructive sleep apnea.

#### Long-acting beta-2 adrenergic agonists (LABA)

In adolescents likewise in adults, the combination of ICS and LABA, including salmeterol and formeterol, improves asthma outcomes more than higher doses of ICS, and it should be considered before increasing the dose of 400 μg BDP or equivalent per day and certainly before administering 800 μg of BDP. A LABA should not be used as monotherapy for asthma but only in fixed-dose combination devices also containing an ICS. Recent studies have shown that the risk of adverse events related to the use of LABA when combined with steroids is similar to the risk of using corticosteroids alone in asthmatic patients (24, 25). However, it is more expensive and does not reduce the risk of further exacerbations compared with ICS alone. In addition to studies reassuring about the use of LABA in adolescent and adults (25), an international trial that followed for 26 weeks 6,208 children, aged 4–11 years, with asthma and an exacerbation in the previous year, has also shown that addition of salmeterol to fixed fluticasone doses is effective and does not lead to increased risk of serious asthma-related adverse outcomes than using the ICS alone (26).

#### Leukotriene receptor antagonists (LTRAs)

LTRAs work by blocking some inflammatory responses, such as tightening of airway muscles and the production of mucus secretion, mediated by leukotriens, which are released during asthmatic reaction by cells involved in the pathogenesis of asthma. In international guidelines LTRA are recommended in monotherapy, as second choice after low dose ICS, for the initial step of chronic asthma treatment. Into the next steps, they are also considered as add-on medications, usually in addition to ICS for improving symptoms and pulmonary function, by an increase in antinflammatory activity (27). They may be considered especially for some patients who experienced side-effects using ICS or children under 5 years old (28), and they are effective especially in exercise-induced asthma (29, 30). A review by Bisgaard et al. on more than 2,700 children and adolescents enrolled in several studies, suggested that the safety profile for montelukast was similar to that of placebo or other usual care therapy, and did not change with long-term use (31).

The options of treatment at **Step 3** differ depending on age group. In young children, a medium dose of ICS plus as-needed SABA is the preferred solution, whereas in adolescents, as well as in adults, the combination of low dose ICS/LABA (BDP/formoterol or budesonide/formoterol) as maintenance treatment with as-needed SABA as reliever, or low dose ICS/LABA (BDP/formoterol) as both maintenance and reliever treatment may be considered. Adding LABA to the same low dose of ICS seem to improve, in this age group, symptoms and lung function and reduce risk of exacerbations, compared with a fixed dose of ICS/LABA as maintenance treatment or a higher dose of ICS, both with as-needed SABA. Another option for adults and adolescents if asthma control remains suboptimal, is to increase ICS to medium dose, but this is less effective than adding a LABA. Other less efficacious options are low dose ICS plus either LTRA or low dose, sustained-release theophylline (3).

For adolescent patients as well as adults, low dose maintenance and reliever ICS/LABA with as-needed SABA, is suggested for **Step 4;** if necessary, in patients with not complete symptoms control, may be considered the use of medium dose ICS. For children <12 years, if asthma control is not achieved using moderate dose ICS, the recommendation is to refer the child for expert assessment. A high dose is recommended only when good asthma control cannot be achieved with medium dose ICS plus LABA and/or a third controller, such as LTRA. However, the increase in ICS dose generally provides little additional benefit, and there is an increased risk of side-effects.

The NICE publication include some conflicting advices on key issues of asthma management in primary care. One controversial difference is about the use of LTRA as first choice add-on therapy for patients whose asthma is not well controlled with low dose ICS. LABA are considered marginally more effective than LTRA, in the analysis by NICE, to justify the additional cost (7). The Primary Care Respiratory Society UK has suggested an initial trial of LTRA as add-on treatment to low dose ICS, but underlie that it should not be changed the treatment of patients already favorably established on a LABA regimen (5).

When patients experience persistent symptoms or exacerbations despite high-dose ICS or ICS/LABA, or other options of Step 4 treatment, there are different pharmacological options that may be considered at **Step 5**, as following listed.

#### Oral corticosteroids

Add-on low dose oral corticosteroids (≤7.5 mg/day prednisone equivalent) may be considered, but may be associated with potential side effects especially in long-term treatments. Prednisolone is the most used steroid for maintenance therapy in patients with chronic asthma (32). Blood pressure, urine or blood sugar, cholesterol, bone mineral density, growth (height and weight centile), should be regularly monitored, and cataracts should be screened in patients using steroid tablets.

#### Omalizumab

Anti-immunoglobulin E (anti-IgE) treatment for patients >12 years of age, with severe allergic asthma, impaired lung function, and proven IgE-mediated sensitivity to inhaled allergens, is also a treatment option. Omalizumab is a humanized monoclonal antibody that interferes with the inflammatory cascade by reducing serum IgE levels and inhibiting IgE binding to receptors. It is given as a subcutaneous injection every 2 or 4 week depending on patient weight and total serum IgE levels. It exhibit a suitable clinical outcome reducing exacerbations, and improving asthma control (33). School-age children and adolescent with moderate-to-severe asthma treated with omalizumab in long-term trials had a significantly reduced number of exacerbation attacks, improved the quality of their life and reduced the need for other standard medications to control asthma (34, 35). Omalizumab is safe and well tolerated but it has the inconvenience of the subcutaneous administration and elevated costs.

#### Immunotherapy

Studies using both subcutaneous and sublingual allergen immunotherapy (SCIT and SLIT) have demonstrated benefit in reducing asthma symptoms and bronchial hyperreactivity in children who do not completely respond to other preventative strategies including ICS (36). The safety profile of SLIT seems to be better than SCIT. There are, however, few studies of adding immunotherapy to ICS so there is difficulty precisely defining where it should sit in step-wise asthma management.

#### Mepolizumab

The use of specific anti-IL5 monoclonal antibodies treatment (mepolizumab), based on eosinophilia (>3%) in induced sputum as biomarkers to identify responsive patients is a debated topic. Some trials indicate that anti-eosinophil biologic drug can be effective for treatment of the severe systemic corticosteroid-dependent asthma, with a “eosinophilic” endotype, whose disease is largely dependent on eosinophil pathophisiology, by a beneficial effect on airway remodeling (37).

## Other pharmacological options

### Theophylline and chromones

Other drug solutions not recommended for routine use are theophylline, the most used methylxanthine, which has low efficacy in asthma bronchoconstriction and common side-effects, especially when administered at higher doses (38), and chromones (nedocromil sodium and sodium cromoglycate) used as mast cell stabilizer, having a suitable safety profile, but low efficacy (39). They are included in guidelines as second-line medications in initial treatment steps and prevention of exercise-induced asthma.

### Intramuscolar triamcinolone

Intramuscolar treatment with triamcinolone has been used for severe asthma treatment. Pediatric studies suggest that it may affect eosinophilic inflammation, improve airway obstruction and prevent exacerbation attacks in children with severe asthma (40, 41), but side effects using it are worse than after treatment with oral corticosteroids (42, 43).

### Anticholinergic agents

The addition of tiotropium bromide, a long-acting inhaled anticholinergic agent, to ICS plus LABA has been shown to improve lung function and asthma symptom control in patients with severe persistent asthma in adults (44). There have been no studies of the use of tiotropium in asthmatic children.

### Metotrexate, antifungal agents, macrolides

There are low evidences in the efficacy of metotrexate in children with asthma. A meta-analysis investigating the steroid-sparing effect of oral metotrexate in asthmatics showed a small benefit despite a high recurrence of adverse effect (45).

Antifungal agents are not recommended in asthmatic patients without allergic broncopulmonary aspergillosis (46). Vicencio and coworkers recently documented that a high percentage of children with refractory asthma had fungal sensitization correlated to the severity of their disease. Based to a case report of a positive response to itraconazole treatment of a child with severe asthma, the Authors suggest that antifungal therapy may represent a potential successful treatment in some patients with evidence of fungal sensitization, after eliminating molds in the environment (47, 48).

Macrolides have been also proposed in combination to the treatment regimen of asthmatic children, with the aim of decreasing the need of the patient's daily dosage of corticosteroids, due to its antinflammatory and antimicrobial activity (49). A recent study including 40 school-aged asthmatic children showed that a 3-week course of clarythromicyn given as add-on therapy to regular treatment, was associated with an increased control of symptoms and a decrease in the duration of the asthma exacerbations (50). Since the use of this antibiotic is safe compared to other medications, it may be indicated in severe asthmatic patients who have mainly neutrophilic airway inflammation and show resistance to other therapy. However, in long term treatment the development of macrolide resistance among respiratory pathogens should be considered.

## Asthma severity assessment

Asthma severity can be assessed when the patient is in a regular control of symptoms with continuous treatment:

- Mild asthma is asthma that is well controlled with Step 1 or Step 2 treatment, i.e., with as-needed reliever medication alone, or with low-dosage controller treatment such as low dose ICS, LTRA, or chromones.
- Moderate asthma is asthma that is well controlled with Step 3 treatment e.g., low dose ICS/LABA.
- Severe asthma is asthma that requires Step 4 or 5 treatment, e.g., high-dose ICS/LABA, to prevent it from becoming “uncontrolled,” or asthma that remains “uncontrolled” despite this treatment. The ERS/ATS Task Force on Severe Asthma has published International guidelines providing definition and guidance about the management of patients with uncontrolled asthma (51).

## Conclusions

We conclude that due to heterogeneity of asthma characteristics, treatment decisions should be critically made, pondering the differences highlighted by agreed international consensus documents.

Symptoms control of asthmatic patients should be closely monitored, as well as risk factors and frequency of exacerbations, and the response to any treatment adjustment should be documented and regularly reviewed by specialists. A step up in treatment may be considered if patients do not respond suitably to initial treatment, after checking for comorbidities, or alternative therapy options may be attempted. A follow-up visit within 1 week after an exacerbation attack should be scheduled, and a written asthma action plan should be completed by the patient as part of a personal asthma management education. An occasional short-term step up for weeks in maintenance pharmacological doses may be necessary, for example, during viral infections or seasonal allergen exposure. Sometimes a daily dose adjustment of the maintenance therapy dosage may be needed according to symptoms. Ongoing monitoring adherence to asthma therapy and asthma control by spirometry in children who can perform it, and self-monitoring at home by peak expiratory flow evaluation, together with avoidance of triggers, is also encouraged.

## Author contributions

RT designed the review and drafted the manuscript. GB, GM, and VM contributed to data collection and interpretation of the literature data. FC revised the work and provided critical input to the manuscript. All Authors approved the final version of the manuscript as submitted.

### Conflict of interest statement

The authors declare that the research was conducted in the absence of any commercial or financial relationships that could be construed as a potential conflict of interest.
